# Pharmacodynamic Evaluation of Phage Therapy in Ameliorating ETEC-Induced Diarrhea in Mice Models

**DOI:** 10.3390/microorganisms12122532

**Published:** 2024-12-08

**Authors:** Yangjing Xiong, Lu Xia, Yumin Zhang, Guoqing Zhao, Shidan Zhang, Jingjiao Ma, Yuqiang Cheng, Hengan Wang, Jianhe Sun, Yaxian Yan, Zhaofei Wang

**Affiliations:** Shanghai Key Laboratory of Veterinary Biotechnology, School of Agriculture and Biology, Shanghai Jiao Tong University, Shanghai 201100, China

**Keywords:** enterotoxigenic *Escherichia coli*, phage, oral therapy, diarrhea, pharmacokinetics

## Abstract

Enterotoxigenic *Escherichia coli* (ETEC) is a major pathogen causing diarrhea in humans and animals, with increasing antimicrobial resistance posing a growing challenge in recent years. Lytic bacteriophages (phages) offer a targeted and environmentally sustainable approach to combating bacterial infections, particularly in eliminating drug-resistant strains. In this study, ETEC strains were utilized as indicators, and a stable, high-efficiency phage, designated vB_EcoM_JE01 (JE01), was isolated from pig farm manure. The genome of JE01 was a dsDNA molecule, measuring 168.9 kb, and a transmission electron microscope revealed its characteristic T4-like Myoviridae morphology. JE01 effectively lysed multi-drug-resistant ETEC isolates. Stability assays demonstrated that JE01 retained its activity across a temperature range of 20 °C to 50 °C and a pH range of 3–11, showing resilience to ultraviolet radiation and chloroform exposure. Furthermore, JE01 effectively suppressed ETEC adhesion to porcine intestinal epithelial cells (IPEC-J2), mitigating the inflammatory response triggered by ETEC. To investigate the in vivo antibacterial efficacy of phage JE01 preparations, a diarrhea model was established using germ-free mice infected with a drug-resistant ETEC strain. The findings indicated that 12 h post-ETEC inoculation, intragastric administration of phage JE01 significantly reduced mortality, alleviated gastrointestinal lesions, decreased ETEC colonization in the jejunum, and suppressed the expression of the cytokines IL-6 and IL-8. These results demonstrate a therapeutic benefit of JE01 in treating ETEC-induced diarrhea in mice. Additionally, a fluorescent phage incorporating red fluorescent protein (RFP) was engineered, and the pharmacokinetics of phage therapy were preliminarily assessed through intestinal fluorescence imaging in mice. The results showed that the phage localized to ETEC in the jejunum rapidly, within 45 min. Moreover, the pharmacokinetics of the phage were markedly slowed in the presence of its bacterial target in the gut, suggesting sustained bacteriolytic activity in the ETEC-infected intestine. In conclusion, this study establishes a foundation for the development of phage-based therapies against ETEC.

## 1. Introduction

*Escherichia coli* (*E. coli*), a Gram-negative bacterium, is commonly found in the intestines of humans and animals [1]. Among its strains, enterotoxigenic *E. coli* (ETEC) is globally recognized as a leading pathogen responsible for intestinal health deterioration and diarrhea in both humans and farm animals [1,2]. ETEC attaches to specific receptors on the apical surface of intestinal epithelial cells via pilus or non-pilus adhesins, subsequently releasing enterotoxicity in the form of ETEC heat-labile toxin (LT) and/or in the form of ETEC heat-stable toxin. There are two types of ST, namely, STa and STb; the two subtypes of STa, namely, STp and STh, are found in ETEC isolated from humans and livestock; in contrast, STb has been found only in ETEC infecting livestock [2]. These toxins disrupt intestinal electrolyte balance, resulting in secretory diarrhea [3]. In livestock, particularly pigs, ETEC is a primary cause of diarrhea in weaned piglets, typically between 18 and 26 days old, leading to postweaning diarrhea (PWD). This condition significantly impairs piglet growth, elevates mortality rates, and causes substantial economic losses in pig farming operations [4,5]. Vaccination, especially a bivalent F4/F18 vaccine, targeting virulence factors such as adhesion factors and STs, remains a primary strategy to prevent ETEC infections in pig farms [5]. However, due to the presence of multiple enterotoxins in most ETEC strains and the lack of coverage of specific strains’ pilus or non-pilus adhesin antigens, commercially available vaccines still offer limited protection [6]. Additionally, while antibiotics are extensively employed to manage ETEC infections, multidrug resistance has escalated. Recent global studies indicate that ETEC strains isolated from diarrheal pigs exhibit widespread resistance to antibiotics such as gentamicin, trimethoprim–sulfamethoxazole, amoxicillin, and ampicillin [7,8,9,10]. Consequently, addressing drug-resistant ETEC infections through the development of novel antimicrobial agents is an urgent priority.

Bacteriophages (phages), viruses capable of targeting and eliminating specific bacterial hosts, are ubiquitously distributed in environments such as water, sediment, soil, sludge, and human mucosal surfaces, making them the most prevalent biological entity on Earth [11,12]. In recent decades, the overuse of antibiotics has accelerated the development of multidrug-resistant bacteria, propelling bacterial therapy into the so-called “post-antibiotic era” [13]. As a natural antimicrobial, phages have garnered increasing attention in clinical settings due to their abundant availability, high bacteriolytic efficacy, and selective action that spares both the human body and beneficial symbiotic bacteria [14]. Phage therapy has shown promising outcomes in treating gastrointestinal infections caused by multidrug-resistant pathogens [15]. Notably, phage cocktail therapy has been shown to mitigate virulence and enhance antibiotic sensitivity in mouse models of enteritis caused by resistant Salmonella strains [16]. For instance, in a mouse colitis model induced by the LF82SK Salmonella strain, associated with Crohn’s disease, EcoActive cocktail therapy demonstrated substantial therapeutic efficacy and a favorable safety profile [17]. Moreover, extensive in vitro research highlights the potential of phages in combating ETEC. Studies report the effective action of phages against ETEC strains that persist in simulated intestinal fluid or Galleria mellonella larvae [18,19]. Despite the numerous reports supporting the efficacy of phage therapy against ETEC infections, comprehensive pharmacological and pharmacokinetic investigations into the treatment of ETEC infections within the animal gastrointestinal tract remain limited.

In this context, a highly effective phage, vB_EcoM_JE01 (JE01), was successfully isolated from a piglet diarrhea sample in this study. The genomic profile, host range, morphological traits, and in vitro stability of JE01 were thoroughly characterized. Its protective effect against ETEC infection in porcine intestinal epithelial cells (IPEC-J2) was confirmed, and its therapeutic efficacy in mitigating ETEC-induced diarrhea in mice was further assessed. Additionally, a fluorescently labeled phage was developed to trace phage metabolism in the digestive tract through in vivo imaging of the mouse intestine. This study offers a robust foundation for the clinical management of piglet diarrhea caused by drug-resistant ETEC and highlights the promising role of phages as viable alternatives to antibiotics in combating resistant bacterial infections.

## 2. Materials and Methods

### 2.1. Statement of Ethics

The experimental procedures involving mice strictly adhered to the Guiding Principles in the Care and Use of Animals (China) and received approval from the Ethical Committee for Animal Experiments of Shanghai Jiao Tong University (China) (Approval no. 202201314).

### 2.2. Identification, Culture, and Drug Resistance Analysis of Bacterial Strains

ETEC strains U74 (GenBank accession number: GCA_017565335.1) and U90 (GenBank accession number: GCA_016696665.1) were kindly provided by Professor Jiale Ma and Professor Huochun Yao, Nanjing Agricultural University, China; both strains were originally isolated from piglet diarrhea in the Jiangsu region of China. The other 23 ETEC strains utilized in this study were sourced from piglet diarrhea samples collected from pig farms in the Shanghai region of China (Table 1). Following isolation, all strains were inoculated in Luria–Bertani (LB) broth and incubated in a temperature-controlled shaker at 37 °C with agitation at 200 rpm. 16S rRNA sequencing was employed to characterize these bacterial samples [20]. Strains identified as *E. coli* through sequencing were further screened for the presence of three ETEC virulence genes, namely, STa, STb, and LT1 (Table 2), using PCR and sequencing. Strains harboring one or more of these genes were classified as ETEC.

Antimicrobial susceptibility testing for clinical enteritis isolates was conducted following Clinical and Laboratory Standards Institute protocol VET01S (CLSI VET01S) 2024 standards, using the Kirby–Bauer disk diffusion method. The antibiotics evaluated included ampicillin (AMP), ciprofloxacin hydrochloride (CIP), gentamicin (GM), meropenem (MEM), and cefepime (FEP). The resistance rate was calculated as the proportion of resistant isolates out of the total number of isolates (Table 1).

### 2.3. Phage Isolation, Purification, and Titer Determination

Phage isolation followed the established protocol of this laboratory [21], utilizing ETEC strains previously isolated from pig farm manure as indicator bacteria. The double-layer agar plate technique [21] was employed for purification and titer determination. The phage stock was serially diluted with 1 × SM buffer, and 100 µL of each phage dilution was combined with 100 µL of indicator bacterial solution. Following a 15-min incubation at 37 °C, the mixture was added to 10 mL of semi-solid LB medium. After thorough mixing, the contents were poured onto solid LB medium plates. Once fully cooled and solidified, plates were inverted and incubated at 37 °C. Plaque counts were conducted after a 12-h incubation. The phage titer (PFU/mL) was calculated as the average plaque count × dilution factor × 10.

### 2.4. Phage Genome Sequencing and Annotation

Purified phage DNA was extracted following the protocol outlined by Wang et al. [21]. Phage whole-genome analysis was performed using shotgun sequencing. Sequence alignments were processed via the Accelrys DS Gene software v2.5 suite (Accelrys Inc., San Diego, CA, USA). Open reading frames were predicted using algorithms from Accelrys Gene v2.5 (Accelrys Inc.) and ORF Finder (NCBI, Bethesda, MD, USA). Identity values were computed through various BLAST algorithms available on the NCBI website (http://www.ncbi.nlm.nih.gov/BLAST/, accessed on 5 May 2023).The sequence of the phage JE01 has been deposited with NCBI (GenBank accession number: PQ471460).

### 2.5. Phage Morphology Analysis via Transmission Electron Microscopy

A 3 μL volume of purified phage suspension was applied to a carbon-coated copper grid. After allowing it to air-dry, the sample was negatively stained with an equal volume of 2% (*w*/*v*) phosphotungstic acid (pH 6.7) for 30 s, ensuring that the staining time did not exceed 1 min. Excess solution was removed with filter paper. Phage particles were then visualized using a Talos L 120C G2 transmission electron microscope (Thermo, Waltham, MA, USA) operated at an accelerating voltage of 120 kV.

### 2.6. Determination of Host Range of Phage

To determine the phage’s host range, 100 µL of suspensions of ETEC strains in the early logarithmic growth phase (optical density at 600 nm, OD600 ≈ 0.5) were mixed with serial tenfold dilutions of phage. The mixture was combined with approximately 10 mL of semi-solid LB medium at 40 °C, thoroughly mixed, and rapidly poured onto solid LB agar plates. Once the agar had solidified, the plates were incubated at 37 °C for 12 h. The phage host range was assessed by the presence of plaques and clear areas indicating bacterial sensitivity.

### 2.7. Assay of Optimal Multiplicity of Infection (MOI) and One-Step Growth

Overnight cultures of ETEC strain U74 were diluted 1:100 in fresh LB and incubated at 37 °C with shaking until the early logarithmic growth phase (OD600 ≈ 0.5), diluted 1:10, and mixed with phages at different MOIs. After 3 h incubation at 37 °C, the mixture was centrifuged at 5000× *g* for 10 min at 4 °C, and the supernatants were filtered through membranes with a pore size of 0.22 μm. The phage titer in the supernatant was immediately determined using a double-layer agar plate method. One-step growth experiments were performed using a modified form of a method described previously [21]. Briefly, JE01 phage was added at an MOI of 1 to the cells of strain U74 and allowed to adsorb for 15 min at 37 °C. The mixture was then centrifuged at 10,000× *g* for 1 min. After the supernatants were removed, the pellets containing the phage-infected bacterial cells were suspended in fresh LB and incubated with shaking at 180 rpm and 37 °C. Partial samples were obtained at 10 min intervals, and the titrations from the aliquots were immediately determined using the double-layer agar plate method. These assays were performed at least in triplicate.

### 2.8. Assessment of Phage Biological Characteristics

The procedures followed were consistent with those previously described [21,22]. For thermal stability analysis, a 1 mL phage suspension at a concentration of 1 × 10^9^ PFU/mL was incubated for 1 h across a temperature range of 20 °C to 80 °C in 5 °C increments. To assess pH stability, the phage suspension (1 × 10^9^ PFU/mL) was incubated in LB broth adjusted to pH 2 to 12 using HCl or NaOH for 1 h. Ultraviolet (UV) sensitivity was evaluated by exposing the phage suspension (1 × 10^9^ PFU/mL) to 30 W UV light at a distance of approximately 35 cm, with 100 μL samples taken every 15 min over a 75-min period. Chloroform sensitivity was tested by incubating the phage suspension (1 × 10^9^ PFU/mL) with various chloroform concentrations (0, 1%, 2%, 5%, 10%, and 20%) at 37 °C for 30 min, followed by centrifugation at 800 rpm for 15 min to separate the hydrophilic layer. Serial dilutions of all phage suspensions were performed using 1 × SM buffer, and titers were determined via the double-layer agar method.

### 2.9. Assay of Phage’s Inhibition of Bacterial Adhesion

An IPEC-J2 culture was obtained from the American Type Culture Collection (ATCC) and maintained in RPMI 1640 medium supplemented with 10% fetal bovine serum (Corning, Corning, NY, USA) at 37 °C in a 5% CO_2_ atmosphere. A strain U74 suspension in the logarithmic growth phase (OD600 ≈ 0.5) was centrifuged at 5000 rpm for 5 min, and the pellet was washed twice with RPMI 1640 medium before resuspension. The OD600 of the suspension was recorded. IPEC-J2 cells (MOI = 1) in a 24-well plate were exposed to 5 × 10^5^ CFU of bacteria per well, followed by centrifugation at 1000× *g* for 10 min at 4 °C and incubation at 37 °C for 1 h in a CO_2_ incubator. The experimental group received 100 μL of phage preparation, while the control group was treated with an equal volume of PBS. After 1 h of incubation, the cells were washed 5–7 times with RPMI 1640 medium. Subsequently, 1 mL of sterile water was added to each well, and after 10 min, the cells were lysed through repeated pipetting and agitation. Bacterial counts were determined by plating serial 10-fold dilutions in sterile water.

### 2.10. Mouse Model

Male BALB/c mice, approximately 5 weeks old, were selected and randomly assigned into groups as outlined in Table 3, with each group consisting of 18 mice. The animals were administered a cocktail of broad-spectrum antibiotics (Abx, metronidazole 1 g/L, neomycin 1 g/L, penicillin 1 g/L, and vancomycin 0.5 g/L) in drinking water for 4 consecutive days to create germ-free conditions. A mouse challenge and phage therapy were performed following the protocol. A suspension of ETEC strain U74, adjusted to an OD value of 0.6, was centrifuged at 5000 rpm for 10 min, followed by two PBS washes to obtain a final concentration of 1 × 10⁹ CFU/mL. A 50 mL solution of phage JE01 (1 × 10⁹ PFU/mL) was prepared, and endotoxins were removed using the ToxinEraser™ Endotoxin Removal Kit (Genscript, Nanjing, China).The endotoxin levels were verified using the ToxinSensor™ Chromogenic LAL Endotoxin Assay Kit to ensure successful removal.

Table 3 outlines the bacterial challenge and phage therapy protocols conducted in mice. Mice were dosed by oral gavage with 50 μL (1 × 10⁹ CFU/mL) ETEC strain U74 suspension or an equivalent volume of PBS. Twelve hours post-inoculation, mice in the phage therapy group were administered 50 μL (1 × 10⁹ CFU/mL) of JE01 via gavage, while those in the antibiotic treatment group received 50 μL (10 mg/kg) of gentamicin (GEN). The PBS control group was similarly gavaged with an equivalent volume of PBS.

To assess disease activity, mice were subjected to daily clinical monitoring, including mortality checks and an adjusted scoring system based on prior methods. The scoring criteria were defined as follows: (1) normal physiology and feces state (score = 0); (2) altered color or consistency of feces (score = 1); (3) liquid feces or the presence of wet tail or mucosal discharge (score = 2); (4) severe diarrhea, poor mental state, or even fatality (score = 3). A score of 1 or higher indicated diarrhea.

### 2.11. Sample Collection and Processing

Mice were sacrificed, and intestinal samples from the jejunum were flushed with saline to clear out the gut contents. A 1.0 cm segment of each jejunum tissue was fixed overnight in 4% paraformaldehyde and subsequently paraffin-embedded for H&E histological analysis [23]. Additionally, two 1.0 cm tissue segments were promptly preserved in 0.5 mL PBS buffer for CFU burden evaluation or in liquid nitrogen for cytokine assays.

### 2.12. CFU Burden in the Jejunum

Fresh intestinal tissues were homogenized in PBS using a cryogenic lapping instrument (Scientz, Ningbo, China). To assess bacterial load, homogenates were subjected to ten-fold serial dilutions and plated on LB agar containing 100 μg/mL of ampicillin, exploiting the inherent ampicillin resistance of ETEC strain U74 (Table 1). Plates were incubated at 37 °C for 18–24 h, after which bacterial counts were recorded as log CFU per intestinal tissue. All measurements were conducted in triplicate.

### 2.13. Cytokine Assays

Total RNA was extracted from IPEC-J2 cells or jejunum tissues using the AllPrep RNA Micro Kit (Qiagen, Hilden, Germany), following the manufacturer’s protocol. cDNA synthesis was carried out with the PrimeScript RT reagent kit (TaKaRa, Dalian, China). mRNA expression was quantified via two-step relative qRT-PCR, using β-actin as the internal reference gene (Table 2). Primer sequences for TNF-α, IL-6, IL-1β, and β-actin are provided in Table 2. Gene expression was normalized to β-actin. Real-time PCR was conducted with the SYBR Premix Ex Taq kit (TaKaRa) on a CFX ConnectTM RT-PCR system (BIO-RAD, Hercules, CA, USA). The 2^−ΔΔCT^ method was employed for mRNA quantification.

### 2.14. Construction of Red Fluorescent Protein-Decorated Phage

The procedures followed were consistent with those that Li et al. described [24]. To generate a phage labeled with red fluorescent protein, the *rfp* gene (RFP forward primer: GTAAGTGGTAAGCTTATGGCCTCCT; RFP reverse primer: TGGTGCTCGAGTCTCAGGAACA) was fused to the COOH terminus of Soc, producing the *rfp*-Soc fragment. This fragment was subsequently transferred into the expression vector pET28a. The fusion construct, RFP-Soc-pET28a, was expressed in *E. coli* B834 (DE3) (EMD, Darmstadt, Germany) and purified using Ni^2+^ affinity chromatography. The recombinant RFP-Soc protein’s purity was verified through SDS-PAGE, which revealed a single prominent band corresponding to a molecular weight of 39.9 kDa, consistent with the expected size of the RFP-Soc fusion protein (Figure S1).

Phage T4 ΔHocSoc from the research group of Pan Tao served as a platform for foreign peptide display on a capsid [24]. *E. coli* B834 (DE3) carrying RFP-Soc-pET28a were cultured in LB medium with ampicillin at 37 °C until they reached an OD600 of 0.8. The cells were then transferred to fresh LB medium supplemented with 0.2 mM IPTG to induce protein expression. Following induction, 10^7^ PFU of Phage T4 ΔHocSoc was introduced to the culture, which was then incubated at 37 °C for 8 h, followed by an additional 3-day incubation at 4 °C. The lysates were subsequently filtered through 0.22 µm sterile filters, and phage titers were determined via the spot plating method. Phage T4 modified with RFP, termed “Phage^RFP^”, was analyzed for fluorescence using the PerkinElmer IVIS optical imaging system (Figure S2). Phage^RFP^ purification followed the size-exclusion chromatography method outlined by LA et al. [25]. The suspension underwent chromatography on a Sepharose 4B column with 0.063 M phosphate buffer (pH 7.2) as the eluent, at a flow rate of 0.3 mL/min. Elution occurred in the highest molecular weight fraction, as verified through fraction titration. Sterile 0.22 µm syringe filters were used for filtration, and the phage titer was quantified using the spot-plate technique.

### 2.15. In Vivo Imaging of Mice

Germ-free mice were established following the protocol described above. After a 24-h fasting period and water deprivation, bacterial gavage was administered as outlined in Table 4, with phage treatment introduced 12 h post-gavage. Mice from each group were randomly selected and euthanized at 15, 30, 45, 90, and 150 min post-treatment, followed by intestinal extraction. Jejunal segments of standardized length were isolated for imaging using the PerkinElmer IVIS in vivo optical imaging system (PerkinElmer, Waltham, MA, USA). Fluorescence was captured over 30 s with an excitation wavelength of 587 nm and an emission wavelength of 610 nm. Image analysis was performed using Living Image software v4.0. The PBS control group served as the background control to adjust for relative fluorescence values across groups, and regions of interest (ROIs) were selected to assess the average fluorescence intensity per unit area, enabling analysis of phage metabolic dynamics within the mouse intestine.

### 2.16. Statistical Analysis

All data were analyzed using analysis of variance (a two-tailed *t*-test or a one-way ANOVA) in GraphPad Prism version 9.0 (GraphPad software Inc., La Jolla, CA, USA); a *p*-value of <0.05 was considered significant.

## 3. Results

### 3.1. Multi-Drug Resistance Profile of ETEC Isolates

PCR and sequencing analyses targeting enterotoxin genes (STa, STb, and LT) were conducted on *E. coli* isolates from piglets with diarrhea. Among them, 25 isolates were confirmed to carry at least one enterotoxin gene, identifying them as ETEC strains (Table 1). These ETEC strains were then subjected to susceptibility testing with five antibiotics: AMP, CIP, GM, MEM, and FEP. As presented in Table 1, 88% (22/25) of the isolates exhibited resistance to AMP, 64% (16/25) to CIP, and 32% (8/25) to GM. All 25 isolates remained susceptible to MEM and FEP.

### 3.2. JE01 Phage Effectively Lysed ETEC Strains

**Figure 1:**
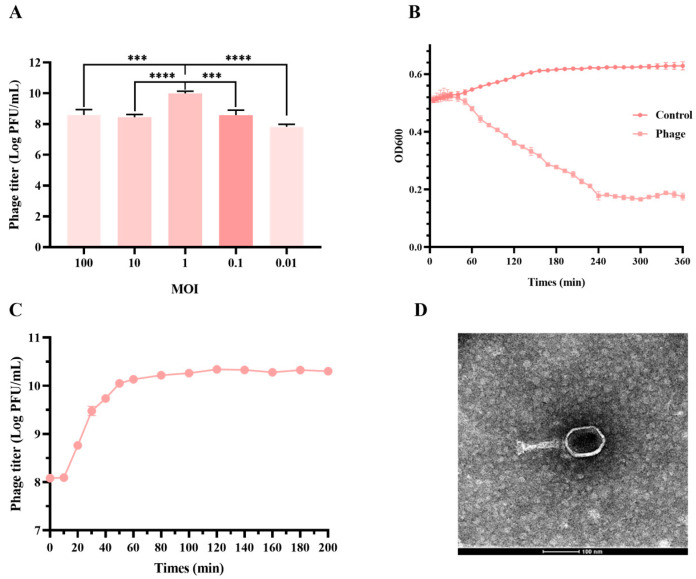
Analysis of the phage JE01′s antibacterial activity. (**A**) Optimal multiplicity of infection (MOI) of JE01. JE01 was used to infect strain U74 at different MOIs, indicating the most suitable concentration for lysing bacteria. (**B**) The in vitro bactericidal effect of the phage JE01. Strain U74 suspensions were mixed with the phage (MOI = 1), and OD600 was measured for 360 min. (**C**) The one-step growth curve of the phage JE01 in ETEC strains U74. The burst size was estimated at 120 PFU per infected cell, which was the ratio of the final count of liberated phage particles to the initial count of infected bacterial cells. (**D**) Transmission electron microscopy of a negatively stained JE01 phage. For (**A**,**B**), experiments were conducted in triplicate, and the results are shown as the mean ± SEM. For (**C**), experiments were conducted in quadruplicate, and the results are shown as the mean ± SEM. For (**A**), statistical significance was determined using one-way ANOVA (Dunnett’s multiple comparisons test) to compare the phage titers between MOI 1 and other MOIs. *** *p* < 0.001, **** *p* < 0.0001.

Manure from pig farms served as the source for phage isolation, with 25 ETEC isolates acting as indicator bacteria for separation using the double-layer plate method. The isolated phage, designated vB_EcoM_JE01 (JE01), exhibited rapid lysis of ETEC strain U74, achieving a titer of about 1×10^10^ PFU/mL (Figure 1A,B). The one-step growth curve of JE01 revealed a short latency period (10 min) and a large burst size (estimated at 120 PFU per infected cell) (Figure 1C). Antimicrobial spectrum analysis revealed that JE01 lysed 36% (9/25) of the ETEC isolates (Table 1). Transmission electron microscopy (TEM) analysis confirmed JE01’s icosahedral morphology, featuring a stereo-symmetric head and a long, contractile tail. The head displayed a regular polyhedral structure with a diameter of 50–60 nm, while the tail measured approximately 100 nm in length and 10 nm in diameter (Figure 1D).

**Figure 2:**
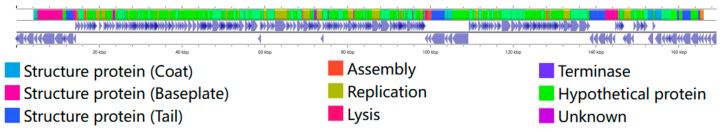
The genomic features of the phage JE01 were determined by making a Proksee map (https://proksee.ca, accessed on 1 November 2023). The functional modules in the phage genomes are shown in different colors.

Phage genome extraction and subsequent whole-genome sequencing revealed that phage JE01 possessed a linear dsDNA genome, spanning 169.123 kb and encoding 263 open reading frames (ORFs). The genome exhibited characteristic modular organization, encompassing regions responsible for DNA replication and modification, structural components, tail structure formation, assembly, and proteins involved in host cell lysis (Figure 2). Notably, no homologous sequences corresponding to toxic genes, antibiotic resistance genes, or lysogeny-associated genes were detected in the JE01 genome. Based on International Committee on Taxonomy of Viruses (ICTV) guidelines and TEM analysis, JE01 was classified within the genus of T4-like viruses in the family Myoviridae.

### 3.3. JE01 Phage Exhibited Better Physicochemical Stability

**Figure 3:**
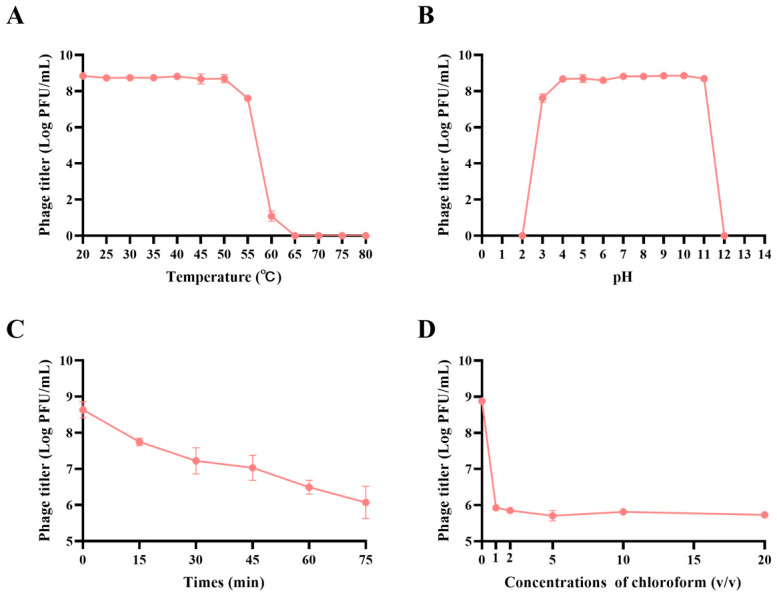
Biological properties of the phage JE01. (**A**) Thermal stability assay: phages were incubated for 1 h at various temperatures ranging from 20 °C to 80 °C at 5 °C intervals. (**B**) pH stability assay: phages were incubated for 1 h at pH values ranging from 2 to 12. (**C**) UV radiation stability assay: phages were exposed to UV light (30 w, 30 cm) for 15, 30, 45, 60, and 75 min. (**D**) Chloroform sensitivity assay: phages were treated with various chloroform concentrations (0%, 1%, 2%, 5%, 10%, and 20%) at 37 °C for 30 min. The surviving phage particles were quantified by double-layer tests. Experiments were conducted in triplicate, and the results are shown as the mean ± SEM.

The results of the JE01 phage’s sensitivity to temperature are shown in Figure 3A. After 1 h of incubation at temperatures between 20 °C and 50 °C, the phage titer remained consistent. However, a slight reduction occurred at 55 °C, and complete inactivation was observed at 60 °C. JE01 also demonstrated broad pH adaptability, maintaining stability at pH values ranging from 3 to 11, with an optimal pH near 8 (Figure 3B). At pH levels below 2 or above 12, phage activity was entirely lost. When exposed to UV light, JE01 retained a degree of stability. As illustrated in Figure 3C, after 15 min of UV exposure, the titer dropped by approximately 10-fold, and by 45 min, the titer had decreased by approximately 100-fold, but even after 75 min, it remained above 10^6^ PFU/mL. Sensitivity to chloroform was analyzed as shown in Figure 3D, which illustrates that JE01′s titer declined by 1000-fold under lower concentrations of chloroform. Nevertheless, at chloroform concentrations up to 20%, the titer stabilized at about 10^6^ PFU/mL, indicating a degree of resilience to chloroform exposure.

Phage genome extraction and subsequent whole-genome sequencing revealed that phage JE01 possessed a linear dsDNA genome, spanning 168.9 kb and encoding 181 open reading frames (ORFs). The genome exhibited a characteristic modular organization, encompassing regions responsible for DNA replication and modification, structural components, tail structure formation, assembly, and proteins involved in host cell lysis (Figure 2). Notably, no homologous sequences corresponding to toxic genes, antibiotic resistance genes, or lysogeny-associated genes were detected in the JE01 genome. Based on International Committee on Taxonomy of Viruses (ICTV) guidelines and TEM analysis, JE01 was classified within the genus of T4-like viruses in the family Myoviridae.

### 3.4. JE01 Phage Alleviated ETEC-Induced Damage to Porcine Intestinal Epithelial Cells

**Figure 4:**
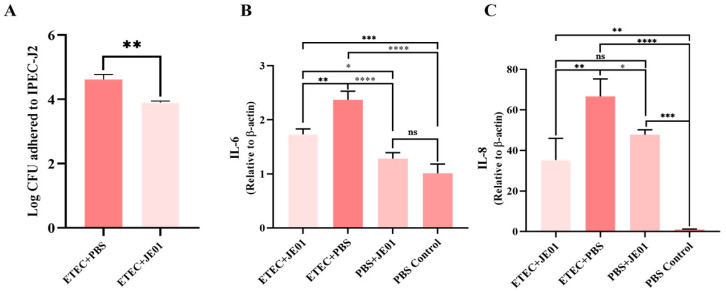
JE01 alleviated ETEC-induced IPEC-J2 cell damage. (**A**) JE01 could alleviate the CFU burden of ETEC adhering to IPEC-J2 cells. (**B**,**C**) JE01 inhibited ETEC-induced production of inflammatory cytokines in IPEC-J2 cells. The levels of the inflammatory cytokines IL-6 and IL-8 were determined. For (**A**–**C**), experiments were conducted in triplicate, and the results are shown as the mean ± SEM. Statistical significance was determined using an unpaired *t* test (**A**) and one-way ANOVA (Tukey’s multiple comparisons test) for multiple comparisons (**B**,**C**). * *p* < 0.05, ** *p* < 0.01, *** *p* < 0.001, **** *p* < 0.0001, ns = not significant.

ETEC adheres to and invades porcine intestinal epithelial cells, triggering an inflammatory response. To assess the potential of JE01 in mitigating ETEC-induced cellular damage, IPEC-J2 cells were first infected with ETEC strain U74 and then treated with JE01. Figure 4A illustrates that after 1 h of incubation, ETEC adhered efficiently to IPEC-J2 cells. In contrast, JE01 treatment (ETEC + JE01) reduced the number of adherent ETEC by about 10-fold compared to the untreated group (ETEC + PBS). Cytokine profiling demonstrated significant reductions in IL-6 and IL-8 levels in JE01-treated cells (after 1 h of incubation), indicating that JE01 effectively suppressed ETEC-induced inflammation in IPEC-J2 cells (Figure 4B,C).

### 3.5. JE01 Phage Effectively Alleviated ETEC-Induced Diarrhea in Mice

**Figure 5:**
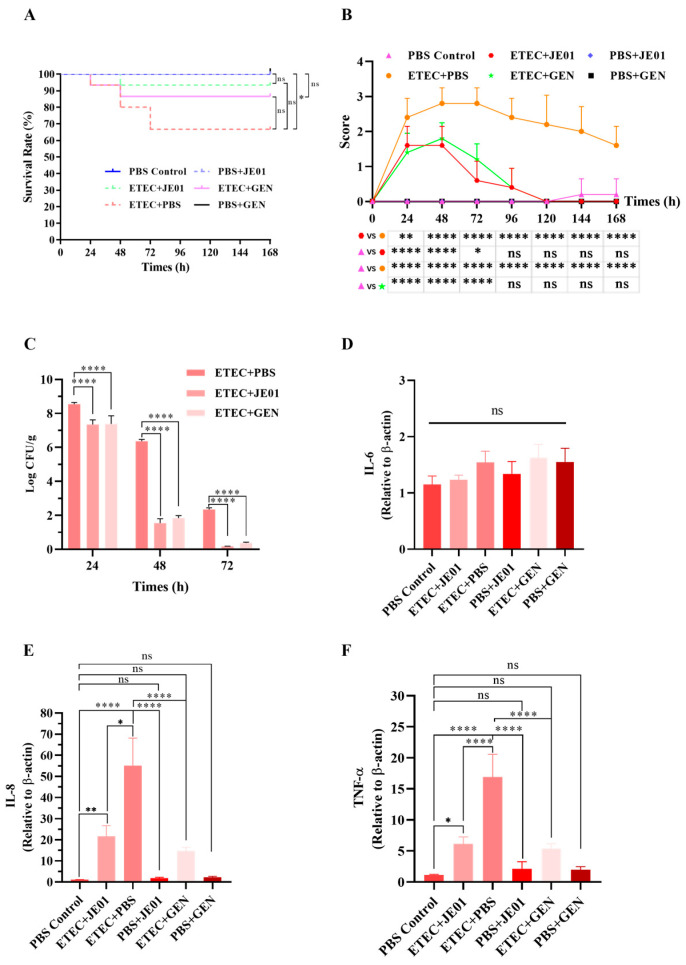
The protective effect of the phage JE01 on an ETEC-induced mouse diarrhea model. Mice that developed diarrhea due to ETEC U74 challenge were dosed by oral gavage with the JE01 phage or GEN, and the survival rate (**A**), diarrhea scores (**B**), and bacterial load of jejunum samples (**C**) were recorded at each of the selected time points. The transcriptional levels of IL-6, IL-8, and TNF-α (**D**) in the jejunum tissues were measured using qRT-PCR. For (**A**), Blue (PBS Control) and black line (PBS GEN) coincided with Blue dotted line (PBS JE01), which of them showed 100% survival. statistical significance was determined using log-rank Mantel–Cox tests for multiple comparisons (*n* = 15/group). For (**B**), data are shown as five murine biological replicates’ mean ± SEM. For (**C**), data are shown as three murine biological replicates’ mean ±SEM at each of the selected time points. For (**D**–**F**), data are shown as three murine biological replicates’ mean ± SEM at 72 h after infection. Statistical significance was determined using one-way ANOVA with Tukey’s multiple comparisons test. * *p* < 0.05, ** *p* < 0.01, **** *p* < 0.0001, ns = not significant.

Fecal samples from Abx-treated mice were screened for the presence of ETEC and phages prior to challenge, revealing no detectable strains of either prior to inoculation. Mice were subsequently orally challenged with ETEC U74, resulting in a survival rate of 66.67% (10/15) within 3 days post-infection (Figure 5A). Clinical symptoms emerged at 12 h post-infection. At this point, mice received treatment with either a phage cocktail or antibiotics (GEN). Phage treatment yielded a survival rate of 93.33% (14/15), while GEN treatment resulted in an 86.67% (13/15) survival rate (Figure 5A). The three control groups—PBS + phage, PBS + GEN, and PBS—achieved 100% survival (Figure 5A). Diarrhea scores indicated that ETEC-infected mice exhibited significantly higher scores on days 1 through 4 post-inoculation than PBS control mice (Figure 5B). However, following oral administration of either phages or GEN, diarrhea scores and clinical symptoms associated with ETEC U74 infection were notably improved (Figure 5B). The therapeutic potential of the phage JE01 in restricting ETEC proliferation during diarrheal episodes were assessed. Consistent with expectations, ETEC-infected mice exhibited elevated pathogen densities in the jejunum (Figure 5C). In contrast, phage- and GEN-treated mice demonstrated markedly reduced ETEC levels in the jejunum at 24 h and 48 h compared to the control groups (Figure 5C), indicating the efficacy of JE01 in mitigating ETEC-induced diarrheal infections. Proinflammatory cytokines are extensively implicated in various pathological processes, including tissue damage and edema, with their levels serving as broad indicators of injury and inflammation [26]. Consistent with this, ETEC markedly elevated the inflammatory markers IL-8 and TNF-α, particularly in the jejunal tissues (Figure 5E,F). Notably, aside from IL-6, a substantial reduction in IL-8 and TNF-α was observed in both the phage and GEN treatment groups (Figure 5E,F). These observations suggest that phage therapy leads to a lower inflammatory response compared to antibiotic treatment. Furthermore, JE01 treatment did not alter cytokine levels in uninfected mice (PBS + JE01 group), confirming the safety and efficacy of JE01 in protecting mice from ETEC infection (Figure 5E,F).

**Figure 6:**
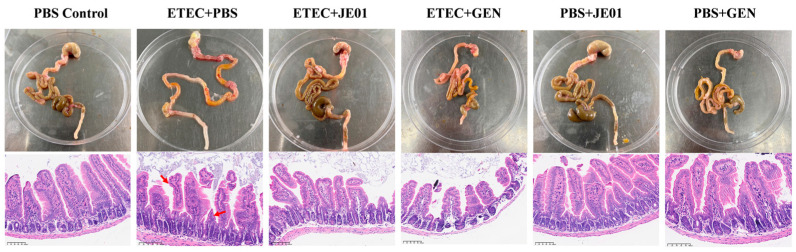
JE01 reduced ETEC-induced intestinal lesions in mice. Mice were infected with ETEC and treated with PBS, JE01, or GEN. Uninfected mice were treated with JE01, GEN, or PBS (PBS Control) and served as controls. Jejunum tissues were collected and processed for H&E staining and microscopic examination. The red arrows indicate villous damage. Magnification: 100×; scale bars: 200 nm.

Pathological changes in the intestines were examined in jejunum tissues stained with H&E at 3 days post-infection. Infected mice displayed marked intestinal wall thinning, gastrointestinal swelling, and hyperemia compared to controls. Severe villous damage in the jejunum was observed following ETEC challenge (Figure 6). Treatment with the phage or GEN significantly restored the gut villi’s integrity, achieving near-complete recovery relative to control groups (Figure 6).

### 3.6. Metabolic Kinetics of the Phage in the Mouse Intestine

**Figure 7:**
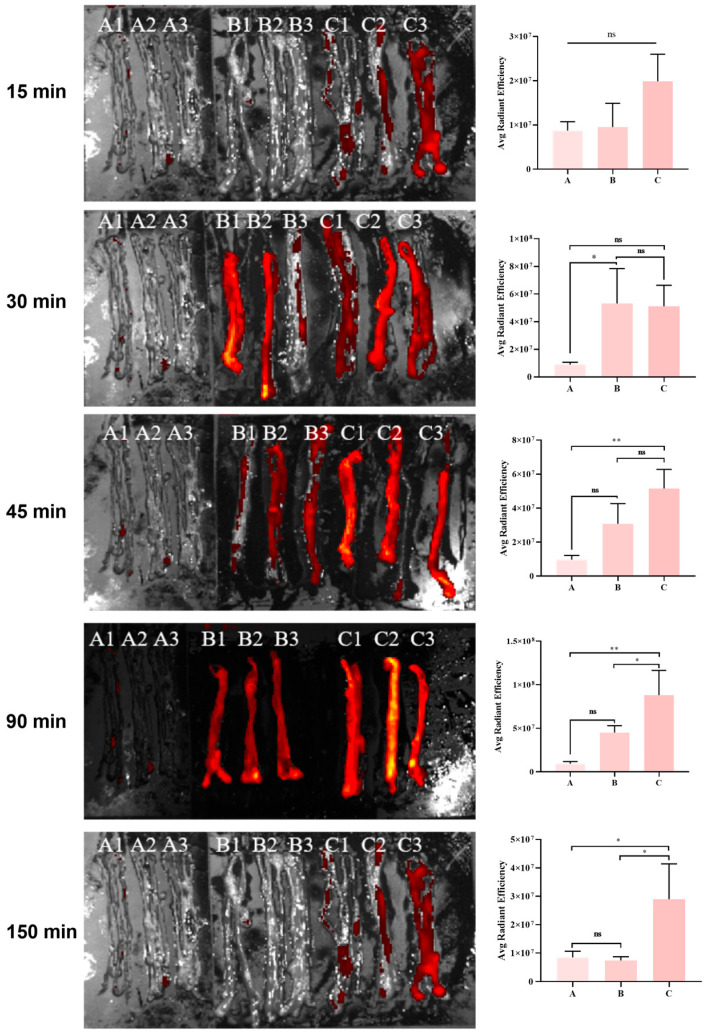
Fluorescence of RFP-displaying phages in the murine jejunum tissues after phage treatment. ETEC-infected jejunum tissues were dissected from mice (N = 3/time points) 15, 30, 45, 90, and 150 min after oral gavage of Phage^RFP^ (Group C). Uninfected mice treated with PBS (Group A) or Phage^RFP^ (Group B) served as controls; Group A served as the background control to adjust for relative fluorescence values across groups, and regions of interest (ROIs) were selected to assess the average fluorescence intensity per unit area. Avg Radiant Efficiency = $\frac{p/\sec/{cm}^{2}/sr}{\mu w/{cm}^{2}}$. Data are shown as three murine biological replicates’ mean ± SEM at every time points. Statistical significance was determined using one-way ANOVA with Tukey’s multiple comparisons test. * *p* < 0.05, ** *p* < 0.01, ns = not significant.

JE01 is a T4-like phage. Research has shown that the T4 phage can utilize its surface capsid structure to display exogenous proteins. In this study, RFP was displayed on the capsid of the T4 ΔHocSoc phage, generating the fluorescent phage (Phage^RFP^). Following ETEC challenge, Abx-treated mice were administered Phage^RFP^. Mice were sacrificed at various time points, and jejunal segments were taken to examine phage metabolism within the intestine. The results indicated that in control group mice, phage retention time in the intestine was brief, with rapid clearance occurring within 150 min (Figure 7). In contrast, phages administered to the challenge group displayed significant intestinal retention by targeting bacteria, leading to markedly increased abundance (Figure 7). These results suggest that the phage can selectively target intestinal pathogens and maintain antibacterial activity, resulting in sustained therapeutic effects.

## 4. Discussion

ETEC is a significant pathogen responsible for diarrhea in children under 5 years old and neonatal animals. It is the primary cause of bacterial diarrhea in piglets, accounting for 65%–70% of all diarrhea-related pathogens [27,28,29]. The high infection rate, morbidity, and mortality, coupled with limited vaccine efficacy and rising antimicrobial resistance, have resulted in substantial economic losses for pig farms worldwide, making ETEC a critical pathogen for farm disease prevention and control [30].

Phages, viruses that infect bacterial cells, have emerged as a promising alternative for controlling ETEC infections on pig farms due to their high specificity, efficacy, abundance, and natural origin [31,32]. Dietary supplementation with phages has been shown to reduce the fecal consistency score (FCS) and increase average daily gain (ADG) in piglets infected with ETEC K88 (F4) [33]. Another study demonstrated that adding freeze-dried phages to feed lowers the ETEC load in the feces of infected piglets [34]. In this study, the phage JE01, isolated from pig farm manure, was found to belong to the Caudovirales, Myoviridae, and exhibited lytic activity against eight distinct ETEC strains, with a broad antibacterial spectrum. Its titer, reaching 10^10^ PFU/mL, surpassed that of other phages reported for ETEC treatment [18,19,20,25]. JE01 also demonstrated exceptional stability across a wide pH range (pH 3-11), maintaining titer levels, which is vital for targeting ETEC colonization in the small intestine, as the phage must withstand stomach acidity (pH~4) to reach and lyse the bacteria [35]. In vitro assays confirmed JE01′s ability to inhibit ETEC adhesion to IPEC-J2 cells, highlighting its therapeutic potential. Furthermore, the safety of phage therapy remains a critical consideration. Whole-genome sequencing revealed no harmful genes, such as those related to toxicity, antibiotic resistance, or lysogeny, in JE01. Additionally, JE01 inhibited the expression of pro-inflammatory cytokines in IPEC-J2 cells, indicating no impact on eukaryotic cells.

To investigate the in vivo antibacterial efficacy of phage JE01, an ETEC-infected mouse model of diarrhea was established for phage treatment. The challenge strain used was the drug-resistant ETEC U74, isolated from diarrheic piglets, with GEN, an antibiotic to which U74 is sensitive, serving as a positive control. Previous studies have demonstrated that 10 mg/kg of GEN effectively treats ETEC-induced diarrhea in mice following oral administration [36]. Consistent with these findings, the current experiment showed that oral GEN administration significantly alleviated diarrhea symptoms in ETEC-infected mice. Notably, JE01 treatment also resulted in substantial recovery of mental state, a marked reduction in intestinal ETEC bacterial load, and significant improvement in diarrhea symptoms. This demonstrates that JE01 exhibits therapeutic efficacy comparable to that of antibiotics in managing ETEC infection, aligning with existing in vivo data on the anti-infective properties of phages and antibiotics.

Extensive literature has documented the use of phage therapy for gastrointestinal diseases and clinical conditions caused by various intestinal pathogens, including adherent–invasive *E. coli* (AIEC), enteroaggregative *E. coli* (EAEC), and drug-resistant Salmonella enteritidis, with substantial therapeutic success reported [16,17,37]. However, most evaluations of phage treatment focus primarily on pharmacodynamics, particularly bactericidal efficacy [38], while pharmacokinetic assessments remain underexplored. Notably, unlike antibiotics, phage pharmacokinetics involve the replication and clearance of phages within the host. Advances in synthetic biology have led to the development of fluorescent phages for pathogen detection, which have become well-established. Their application in animal models during treatment presents a valuable tool for tracing and investigating the metabolic dynamics of phage preparations [39,40]. To preliminarily assess the pharmacokinetics of phage, the T4ΔHocSoc phage display platform was employed to present RFP on the phage capsid, resulting in red fluorescent phages capable of emitting detectable signals. Mouse intestinal imaging experiments demonstrated that the phage reached the intestine 45 min after oral gavage, with fluorescence intensity diminishing at 90 and 150 min. Notably, from 45 min onward, a marked difference in fluorescence intensity was observed between the phage-treated group inoculated with bacteria and the control group that received only phage gavage. In the same unit area of the small intestine, fluorescence was more pronounced in the treatment group. As the experiment progressed to 90 and 150 min, this difference became increasingly evident. Although both groups exhibited a decline in fluorescence intensity, the rate of decrease in the treatment group was significantly slower. At 150 min, imaging revealed a substantial reduction in residual phages in the intestines of the control group, with most phages already excreted, whereas in the treatment group, phage accumulation remained significant. These findings provide compelling evidence of the phage’s rapid targeting ability and sustained bacteriolytic activity in the jejunum infected with ETEC, offering preliminary insight into the potential clinical application of phage preparations for treating intestinal infections such as diarrhea, particularly from a pharmacokinetic perspective.

## 5. Conclusions

ETEC is a significant pathogen affecting intestinal health in both humans and livestock, particularly leading to diarrhea in children, piglets, calves, and lambs. Its impact can lead to severe public health challenges and substantial economic losses for farms. The limited efficacy of vaccines, coupled with rising antibiotic resistance, presents a major barrier to managing ETEC infections. Given the numerous benefits of phage therapy, it has emerged as a promising alternative or adjunct to antibiotics, with considerable potential for broader application. This study developed the antibacterial preparation JE01 and assessed its safety and therapeutic efficacy in IPEC-J2 cells and ETEC-infected mouse enteritis models. A fluorescent phage was engineered using synthetic biology techniques, enabling in vivo fluorescence molecular imaging of the mouse intestines, which was used to analyze the metabolic dynamics of the phage during treatment. These results demonstrate that JE01, as an orally administered antibacterial agent capable of directly targeting the intestine, holds significant potential for combating ETEC infections. Pharmacokinetic imaging of the mouse intestine clearly illustrated that the phage rapidly targeted pathogens and sustained its therapeutic effects throughout treatment. This provides an initial theoretical foundation for understanding the pharmacokinetic mechanisms underlying phage-based strategies for preventing and controlling intestinal bacterial infections.

Phage therapy holds significant potential but faces several key challenges. First, the rapid evolution of bacteria can lead to resistance against phages, necessitating the ongoing discovery of new phages to stay ahead of bacterial adaptation. Second, regulatory obstacles and the absence of standardized production methods complicate the transition of phage-based therapies from research to clinical use. Finally, the variability in human immune responses, coupled with the complex interactions among phages, bacteria, and the host, underscores the need for further research to ensure the safety and effectiveness of these therapies across humans and animals.

## Figures and Tables

**Table 1:** PCR identification, drug resistance, and phage susceptibility of ETEC strains.

ETEC Strains	Enterotoxin			Drug Resistance					Phage
	STa	STb	LT1	AMP (R: 88% ^a^)	CIP (R: 64%)	GM (R: 32%)	MEM (R: 0%)	FEP (R: 0%)	JE01
U74	+ ^b^	−	−	R ^c^	S	S	S	S	+
U86	−	+	+	R	S	R	S	S	−
XH12	+	+	−	R	I	S	S	S	+
XH18	−	−	+	R	S	R	S	S	−
XH22	−	+	+	R	R	R	S	S	+
K88-4	+	+	+	I	S	S	S	S	−
K99-1	+	−	−	S	S	S	S	S	+
NJ-08	+	+	+	I	I	S	S	S	+
NJ-09	+	+	−	R	S	S	S	S	−
NJ-14	−	+	+	R	R	R	S	S	+
NJ-18	−	+	+	R	R	I	S	S	−
NJ-24	+	+	−	R	R	R	S	S	−
NJ-26	+	+	+	R	R	S	S	S	−
NJ-28	+	+	−	R	I	S	S	S	+
NJ-30	+	+	+	R	R	I	S	S	+
MS-04	+	+	−	S	S	S	S	S	−
MS-12	+	+	−	I	I	S	S	S	−
MS-14	+	+	−	R	R	R	S	S	−
MS-27	−	−	+	R	S	S	S	S	−
MS-31	+	+	−	R	R	S	S	S	−
JD-12	+	+	−	S	S	S	S	S	−
JD-23	+	−	−	R	R	S	S	S	−
JD-27	−	−	+	R	I	S	S	S	−
JD-32	+	−	−	R	R	S	S	S	+
JD-38	−	−	+	I	I	S	S	S	−

^a^ Percentage represents the proportion of antibiotic-resistant strains among the total of 25 ETEC strains. + ^b^ indicates positive PCR or JE01 phage-sensitive strains; − indicates negative PCR or JE01 phage-insensitive strains. ^c^ R, drug resistance; S, drug sensitivity; I, intermediate drug resistance. AMP = ampicillin (10 μg/mL), CIP = ciprofloxacin (5 μg/mL), GM = gentamicin (10 μg/mL), MEM = meropenem (10 μg/mL), FEP = cefepime (30 μg/mL).

**Table 2:** Primers used for this study.

Primer	Primer Sequences	
STa-F	GGGTTGGCAATTTTTATTTCTGT	
STa-R	ATTACAACAAAGTTCACAGCAGTA	
STb-F	ATGTAAATACCTACAACGGGTGAT	
STb-R	TATTTGGGCGCCAAAGCATGCTCC	
LT1-F	TAGAGACCGGTATTACAGAAATCTGA	
LT1-R	TCATCCCGAATTCTGTTATATATGTC	
Porcine IL-8-F	TAGGACCAGAGCCAGGAAGA	
Porcine IL-8-R	AGCAGGAAAACTGCCAAGAA	
Porcine IL-6-F	CCTCTCCGGACAAAACTGAA	
Porcine IL-6-R	TCTGCCAGTACCTCCTTGCT	
Porcine β-Actin-F	GGACTTCGAGCAGGAGATGG	
Porcine β-Actin-R	GCACCGTGTTGGCGTAGAGG	
Murine IL-8-F	TCGAGACCATTTACTGCAACAG	
Murine IL-8-R	CATTGCCGGTGGAAATTCCTT	
Murine IL-6-F	CTGCAAGAGACTTCCATCCAG	
Murine IL-6-R	AGTGGTATAGACAGGTCTGTTGG	
Murine TNF-α-F	CAGGCGGTGCCTATGTCTC	
Murine TNF-α-R	CGATCACCCCGAAGTTCAGTAG	
Murine β-Actin-F	AAGAGCTATGAGCTGCCTGA	
Murine β-Actin-R	TACGGATGTCAACGTCACAC	

**Table 3:** Experimental therapeutic grouping in a mouse model of diarrhea.

Group (*n* = 24)	Bacterial Challenge	Treatment
PBS Control	50 μL PBS	50 μL PBS
ETEC + PBS	50 μL ETEC	50 μL PBS
ETEC + JE01	50 μL ETEC	50 μL JE01
ETEC + GEN	50 μL ETEC	50 μL GEN
PBS + JE01	50 μL PBS	50 μL JE01
PBS + GEN	50 μL PBS	50 μL GEN

**Table 4:** Grouping of mice intestinal imaging.

Group (*n* = 15)	Bacterial Challenge	Treatment
PBS control	PBS	PBS
PBS + Phage^RFP^	PBS	Phage^RFP^
ETEC + Phage^RFP^	ETEC	Phage^RFP^

## Data Availability

The original contributions presented in the study are included in the article and supplementary material, further inquiries can be directed to the corresponding author.

## Supplementary Materials

The following supporting information can be downloaded at: https://www.mdpi.com/article/10.3390/microorganisms12122532/s1, Figure S1: Protein profiles of RFP-Soc extracts from expression bacterial strains. Figure S2: Fluorescence assay of Phage^RFP^.
