# Supplementary material for: Pharmacodynamic Evaluation of Phage Therapy in Ameliorating ETEC-Induced Diarrhea in Mice Models
Source: Microorganisms. 2024 Dec 8;12(12):2532. doi: 10.3390/microorganisms12122532 (PMC11678793; doi:10.3390/microorganisms12122532)
Supplement: Supplementary file 1 [file microorganisms-12-02532-s001.zip › microorganisms-3316604-supplementary.pdf]

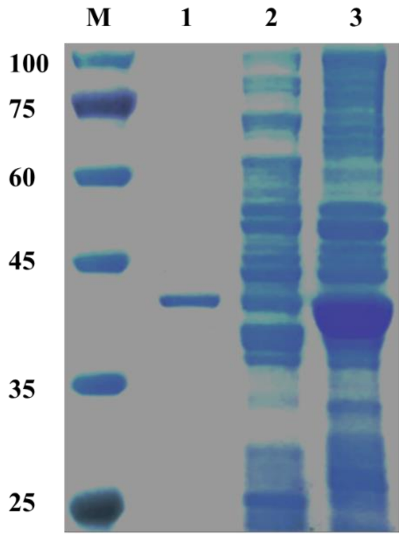

Figure S1: Protein profiles of RFP-Soc extracts from expression bacterial strains. Every lane was added with 10μl total volume. Lane M: Marker. Lane 1: the purified RFP-Soc protein (about 39.9 kDa). Lane 2: the empty vector control. Lane 3: the unpurified RFP-Soc protein.

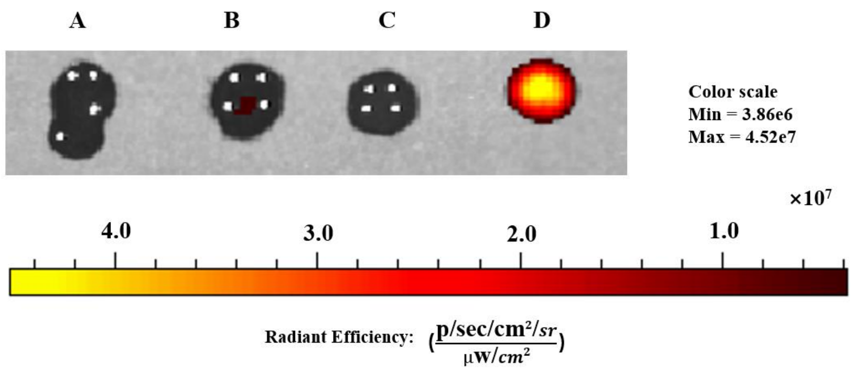

Figure S2: Fluorescence assay of Phage^{RFP}. Every spot represented a drop of 5ul phage. A: T4 phage. B: The progeny phage was derived from *E. coli* B834(DE3) carrying RFP-Soc-pET28a infected by T4 phage. C: T4 ΔHocSoc phage. D: The progeny phage was derived from *E. coli* B834(DE3) carrying RFP-Soc-pET28a infected by T4 ΔHocSoc phage (Phage^{RFP})
